# Analytical method for determining the sulfur content of EP (extreme pressure) additives in lubricating oils

**DOI:** 10.1016/j.mex.2026.103909

**Published:** 2026-04-10

**Authors:** Roland Nagy, Petra Kolnhófer, Gábor Zoltán Nagy

**Affiliations:** University of Pannonia, Faculty of Engineering, Department of MOL Hydrocarbon and Coal Processing, Egyetem u. 10., Veszprém, H-8200, Hungary

**Keywords:** Lubricant additives, Extreme pressure additives, Sulfur content determination, Sulfurized vegetable oils, UV–VIS spectrophotometry, EDXRF

## Abstract

Sulfur-containing organic compounds are important Extreme Pressure (EP) additives used in lubricants and are typically produced by the sulfurization of unsaturated organic compounds. The two most widely applied sulfurization routes—dark and light—result in products with markedly different properties. Dark sulfurization yields less stable additives with unpleasant odour, whereas light sulfurization technologies produce brighter and more stable products with superior performance, albeit at higher production costs. In response to increasing sustainability requirements, sulfurized vegetable oils have gained increasing attention as EP additives due to their renewable origin and biodegradability.

Accurate determination of sulfur content is essential for the development, optimization, and quality control of EP additives. Various analytical methods are available for sulfur determination in the oil industry; however, many of them require expensive instrumentation or complex sample preparation.

In this work, a UV–VIS spectrophotometry-based method was developed and evaluated for the determination of sulfur content in sulfur-containing EP additives. The method was validated by comparison with standard methods (ASTM D 6481 and ASTM D 5185), showing good agreement at 312 nm.

• This method applies UV–VIS spectrophotometry to determine the sulfur content of EP additives.

• This method offers a rapid and cost-effective alternative to widely used methods for the determination of sulfur content in industrial EP additives and lubricating oils.

## Graphical abstract

**Figure:**
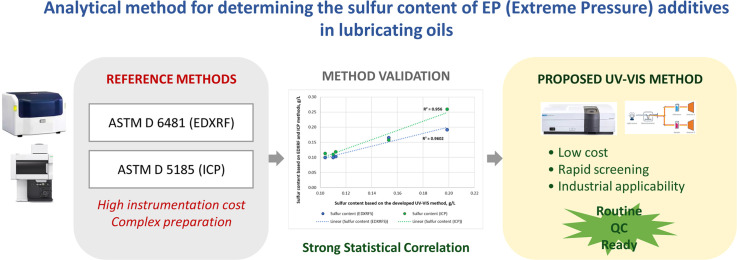

## Specifications table

**Table:** 

**Subject area**	Materials Science
More specific subject area	Analytical chemistry in petroleum industry
**Name of your method**	Analytical method for determining the sulfur content of EP (Extreme Pressure) additives in lubricating oils
**Name and reference of original method**	
**Resource availability**	

## Background

The primary motivation for detailing this methodology lies in the critical need for standardized, high-precision analytical frameworks within the lubricant industry, particularly concerning Extreme Pressure (EP) additives. As environmental regulations—such as those established by the European Union—become increasingly stringent regarding sulfur content and toxic by-products (e.g. dioxins from chlorinated paraffins), the development and accurate characterization of sustainable alternatives have become paramount.

This section provides readers with the necessary context to understand why a multi-faceted analytical approach is required for modern lubricant chemistry. The chemical complexity of petroleum-based matrices and the emergence of bio-based substitutes, such as sulfurized vegetable oils (e.g. rapeseed, soybean), necessitate a robust methodology that can distinguish between various sulfur species. For instance, the performance of an EP additive is not solely dependent on total sulfur concentration but also on the specific chemical bonds present, such as the distinction between active polysulfide bridges and inactive stable carbon-sulfur bonds.

The methodology described herein may be used to:

1. Optimize additive synthesis: By employing techniques such as NMR and IR spectroscopy, researchers can monitor the efficiency of "light" versus "dark" sulfurization processes, ensuring the production of high-quality, odourless, and thermally stable additives.
2. Ensure regulatory compliance: The integration of UV fluorescence and oxidative micro-coulometry allows for the detection of sulfur at sub-ppm levels, which is essential for meeting environmental standards and preventing catalyst poisoning in refinery processes.
3. Characterize performance-structure relationships: The use of hyphenated techniques, such as HPLC-ICP-MS/MS and GC-SCD, provides a granular view of molecular structures like Zinc Dialkyl Dithiophosphate (ZDDTP). This allows for a better understanding of how alkyl chain lengths influence thermal and hydrolytic stability, directly impacting the anti-wear and EP properties of the final product.

This methodology adds significant value to the field of tribochemistry by documenting these procedures. It offers a comparative overview of traditional "bomb methods" (ASTM D129) and modern spectroscopic techniques, enabling researchers and quality control professionals to select the most appropriate analytical path based on their specific matrix and concentration requirements. Ultimately, this framework facilitates the transition toward environmentally friendly, high-performance lubrication technologies [[1], [2], [3], [4], [5], [6], [7], [8], [9], [10], [11], [12], [13], [14], [15], [16], [17], [18], [19], [20]].

## Method details

### Equipment and materials

UV–VIS spectrophotometric measurements were performed using an Avantes AvaSpec-DUAL StarLine fiber-optic spectrometer equipped with a symmetric Czerny–Turner monochromator. The instrument allows independent configuration of two channels; measurements were performed using a single optimized channel.

Illumination was provided by an Avantes AvaLight-D(H)-S combined deuterium–halogen light source covering the 190–2500 nm spectral range. Prior to measurements, the light source was allowed to warm up for at least 20–30 min to ensure intensity stabilization.

Spectral measurements were carried out using a quartz cuvette (10 mm optical path length, 3.8 mL volume) to ensure transparency in the UV range.

#### Materials

• Spectrophotometer: Avantes AvaSpec-DUAL StarLine
• Light source: Avantes AvaLight-D(H)-S
• Cuvette: quartz, 10 mm path length
• Solvent: butoxyethanol (used for dilution and blank)
• Samples: sulfurized vegetable oils (e.g., FAME-based, olive pomace oil)
• Reference: elemental sulfur

### Sample preparation

• Sulfurized oil samples were weighed and dissolved in butoxyethanol to obtain a final concentration of **1 g/L**.
• The solutions were mixed using **magnetic stirring for approximately 5 min** to ensure complete dissolution.
• Prepared samples were allowed to equilibrate at **room temperature (25 ± 2°C)** for at least **5 min** before measurement.
• Calibration solutions were prepared using sulfurized FAME samples with known sulfur content in the range of **15–23 wt%**.

### Step-by-step measurement procedure

• Instrument preparation

1. The spectrometer and light source were switched on and allowed to stabilize for 20–30 min.

• Blank measurement

1. A blank spectrum was recorded using butoxyethanol in a quartz cuvette.
2. This spectrum was used for baseline correction.

• Cuvette handling

1. The cuvette was rinsed with the sample solution prior to each measurement.
2. The cuvette was filled carefully to avoid air bubbles.

• Spectral acquisition

1. Spectra were recorded in the range of 250–400 nm.
2. Quantitative evaluation was performed at 312 nm, where the highest and most stable absorbance was observed.
3. Measurements at 360 nm were also recorded for comparison purposes.

• Replicate measurements

1. Each sample was measured three times (n = 3) under identical conditions.
2. The average absorbance value was used for calculations.

• Temperature conditions

1. All measurements were performed at 25 ± 2°C without additional temperature control.

### Matrix correction and background handling

Due to the intrinsic color of sulfurized oil matrices, especially for dark sulfurized products, background absorption may interfere with the analytical signal.

To correct for this:

• A solvent blank (butoxyethanol) was applied for baseline correction.
• When necessary, matrix-matched blank measurements were used to account for intrinsic oil absorbance.
• All spectra were corrected by subtracting the corresponding blank spectrum.

Sample dilution (1 g/L) was selected to ensure that absorbance values remained within the linear range while minimizing matrix-related interference.

### Statistical evaluation and calibration

The relationship between absorbance and sulfur content was determined using linear regression:

y=ax+b

Calibration equations:

$$312\;\text{nm}\left(\text{Case}\;A\right):y=4.848x+1.2673\left(R^{2}=1.0000\right)$$

$$360\;\text{nm}\left(\text{Case}B\right):y=2.0781x+0.3983\left(R^{2}=0.9992\right)$$

The acceptable confidence interval of the measurement results was within **±5%**.

### Effect of impurities and matrix components

The potential influence of impurities on the measurement accuracy was considered. Since the method is based on UV absorption, any components absorbing in the same spectral region may interfere with the analytical signal.

In sulfurized vegetable oil systems, the main potential interferents include unsaturated compounds and colored degradation products. However, the use of matrix-matched calibration minimizes these effects, as both calibration standards and samples share similar chemical composition.

As a result, no significant impact of impurities was observed within the investigated sample set. Nevertheless, the applicability of the method is limited to matrices similar to those used for calibration.

### Chemical basis, selectivity, and detection mechanism

The method is intended for the determination of total sulfur content in sulfurized organic additives.

The analytical signal arises from the UV absorption of sulfur-containing organic species (e.g., sulfides and polysulfides), which exhibit characteristic electronic transitions in the UV region.

Among the investigated wavelengths, 312 nm provided the most intense and stable signal, and was therefore selected for quantitative analysis.

The method is based on calibration using sulfurized reference samples; thus, the absorbance measured at 312 nm correlates with the total sulfur content of the system.

Although different sulfur species may contribute differently to the absorbance, matrix-matched calibration ensures reliable determination within the defined application range.

### Method advantages

• Rapid and cost-effective analysis
• Minimal sample preparation
• No need for hazardous reagents
• Suitable for routine industrial applications

### Environmental aspects and greenness of the method

The method does not require toxic solvents such as pyridine, benzene, or chlorinated compounds.

Sample preparation involves only butoxyethanol and requires low reagent consumption. No hazardous by-products are generated.

Therefore, the method is consistent with the principles of **green analytical chemistry** and aligns with the sustainability focus of sulfurized vegetable oil-based additives.

## Method validation

### Determination of sulfur content

The sulfur content of the samples was determined using calibration curves. Calculations were performed at two wavelengths: 312 nm (Case A) and 360 nm (Case B). For the determination at 312 nm, the following linear regression equation was applied:

y=4.848x+1.2673

x=(y−1.2673)/4.848

Where “y” is the measured absorbance and “x” is the calculated sulfur concentration.

### Comparison with reference methods

The accuracy of the spectrophotometric method was validated against two standard methods:

ASTM D 6481-14 (EDXRFS) (Table 1) and ASTM D 5185-18 (ICP) (Table 2).

**Table 1:** Comparison of calculated sulfur content with ASTM D 6481-14 (EDXRFS) results.

	Sulfur content (based on the developed UV-VIS method), g/L		Sulfur content (based on ASTM D 6481-14 method), g/L	Difference [%]	
	Case A	Case B		Case A	Case B
**SVO_sample-01**	0.1986	0.8814	0.1921	3.37	358
**SVO_sample-02**	0.1532	0.7756	0.1655	-7.43	368
**SVO_sample-03**	0.1119	0.6793	0.1034	8.26	556
**SVO_sample-04**	0.1099	0.6745	0.1000	9.88	574
**SVO_sample-05**	0.1037	0.6601	0.1000	3.69	560

**Table 2:** Comparison of calculated sulfur content with ASTM D 5185-18 (ICP) results.

	Sulfur content (based on the developed UV-VIS method), g/L		Sulfur content (based on ASTM D 5185-18 method), g/L	Difference [%]	
	Case A	Case B		Case A	Case B
**SVO_sample-01**	0.1986	0.8814	0.2597	-25.50	239
**SVO_sample-02**	0.1532	0.7756	0.1576	-2.79	392
**SVO_sample-03**	0.1119	0.6793	0.1187	-5.71	472
**SVO_sample-04**	0.1099	0.6745	0.1104	-0.50	510
**SVO_sample-05**	0.1037	0.6601	0.1135	-8.63	481

Based on the results, Case B (360 nm) showed significant deviations from the reference methods, likely due to high measurement variance at that wavelength. Therefore, further validation focuses on Case A (312 nm).

Significant deviations were observed when the sulfur content was calculated at 360 nm compared to both ASTM reference methods. This can be attributed to the lower sensitivity and higher signal variability at this wavelength.

At 360 nm, the absorbance of sulfur-containing species is weaker and more susceptible to background interference from the oil matrix, resulting in a reduced signal-to-noise ratio. In contrast, at 312 nm, the absorbance is stronger and more stable due to more pronounced electronic transitions of sulfur-containing chromophores.

Therefore, the method shows improved accuracy and reliability at 312 nm, and this wavelength was selected for further validation and quantitative evaluation.

### Statistical analysis and correlation

**Fig. 1:**
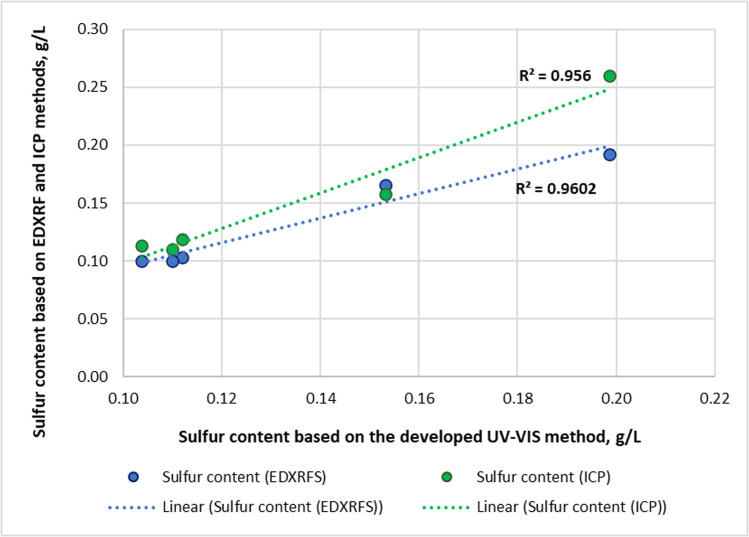
Comparison of calculated results (Case A) with EDXRFS and ICP methods.

The correlation between the calculated results (Case A) and the reference methods is shown in Fig. 1.

The results obtained via spectrophotometry (Case A) have a good correlation with the widely used industrial standard methods.

The results obtained by spectrophotometry (Case A) showed strong correlation with the industrial standard methods (R² = 0.9602 for ASTM D 6481 and R² = 0.9560 for ASTM D 5185). Since correlation alone does not demonstrate analytical agreement, paired t-test and Bland–Altman analyses were also performed.

### Statistical comparison with reference methods

To further evaluate the agreement between the developed UV–VIS method (Case A, 312 nm) and the standard reference methods, paired t-tests were performed at a 95% confidence level. For the comparison with ASTM D 6481, the calculated t-value was 0.810 with a p-value of 0.464, while for ASTM D 5185 the calculated t-value was −1.469 with a p-value of 0.216. In both cases, the p-values were higher than 0.05, indicating that no statistically significant difference was observed between the proposed UV–VIS method and the reference methods.

In addition, Bland–Altman analysis was used to assess method agreement. For ASTM D 6481, the mean bias was 0.00326 g/L, with limits of agreement from −0.01439 to 0.02091 g/L. For ASTM D 5185, the mean bias was −0.01652 g/L, with limits of agreement from −0.06582 to 0.03278 g/L.

These results indicate acceptable agreement between the developed UV–VIS method and the standard ASTM methods over the investigated concentration range.

### Analytical figures of merit

The limit of detection (LOD) and limit of quantitation (LOQ) were calculated based on the standard deviation of the blank signal and the slope of the calibration curve. For the method at 312 nm, the LOD and LOQ were determined to be 0.0020 g/L and 0.0062 g/L, respectively, confirming the high sensitivity of the method for sulfur determination in EP additives.

The working detection range of the method was defined by the calibration standards and corresponds to sulfur contents in the range of 15–23 wt%, which represents the typical concentration range of industrial EP additives.

### Precision of the method

The precision of the method was evaluated under repeatability conditions by performing triplicate measurements (n = 3) for each sample. The precision was expressed as relative standard deviation (RSD, %).

The obtained RSD values were below 5%, indicating good repeatability of the method for sulfur determination in EP additives.

## Limitations

Not applicable.

## Ethics statements

Not applicable.

## CRediT authorship contribution statement

**Roland Nagy:** Conceptualization, Writing – review & editing, Supervision, Project administration. **Petra Kolnhófer:** Investigation, Methodology, Validation, Writing – original draft. **Gábor Zoltán Nagy:** Visualization, Investigation, Resources, Writing – original draft.

## Declaration of competing interest

The authors declare that they have no known competing financial interests or personal relationships that could have appeared to influence the work reported in this paper.

## Data Availability

No data was used for the research described in the article.
